# Decellularized Cell‐Secreted Extracellular Matrices as Biomaterials for Tissue Engineering

**DOI:** 10.1002/smsc.202400335

**Published:** 2024-12-06

**Authors:** David H. Ramos‐Rodriguez, J. Kent Leach

**Affiliations:** ^1^ Department of Orthopaedic Surgery UC Davis Health Sacramento CA 95817 USA; ^2^ Department of Biomedical Engineering University of California Davis CA 95616 USA

**Keywords:** biomaterial, decellularization, extracellular matrix, tissue engineering

## Abstract

The extracellular matrix (ECM) is the naturally secreted biomaterial scaffold that provides support and regulates key aspects of cell behavior. This dynamic and complex network of structural proteins, proteoglycans, and soluble cues defines the cell microenvironment and is essential for tissue homeostasis. Because tissue engineering approaches aim to recapitulate aspects of the microenvironment to instruct tissue regeneration, ECM‐inspired or ‐derived scaffolds are some of the earliest tissue‐engineered constructs reported. However, conventional single‐protein constructs fail to provide the biochemical and structural complexity of the native ECM. Decellularized ECM is under investigation to improve cell adhesion, cell remodeling, migration, proliferation, and differentiation within tissue‐engineered constructs. However, challenges associated with poor mechanical properties and inherent chemical instability compared to synthetic or other natural polymers require additional considerations. This review describes the bioactive properties of ECM, current strategies to efficiently decellularize cell‐secreted and tissue‐derived ECM, standard fabrication techniques for ECM constructs, and current developments in the field of ECM‐based musculoskeletal platforms.

## Graphical abstract

The extracellular matrix (ECM) is a naturally secreted biomaterial scaffold that provides support and regulates key aspects of cell behavior. This review describes the bioactive properties of ECM, current strategies to decellularize cell‐secreted and tissue‐derived ECM, standard fabrication techniques for ECM constructs, and current developments in the field of ECM‐based musculoskeletal platforms.

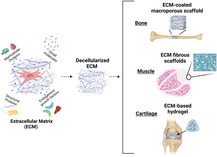

## 1 Introduction

The extracellular matrix (ECM) is a complex network of macromolecules that is secreted by all cells and includes the non‐cellular components of tissues and organs.^[^ ^1^ ^]^ It represents the biochemical and physiological cues (also denoted as matrisome) that define the cellular microenvironment, governs cell fate, and provides physical support to tissues while acting as a reservoir of growth factors that control survival, differentiation, and migration.^[^ ^2^ ^]^ The ECM is a dynamic network that is constantly remodeled by surrounding cells via interaction with integrin and non‐integrin surface receptors and intracellular signaling cascades.^[^ ^3^ ^]^ Furthermore, its composition, organization, and function are tissue‐specific and dependent on the resident cell population. Tissues such as cartilage and skin rely on the spatial and biochemical hierarchies of ECM for their mechanical function and to control cell differentiation and function, while trabecular bone marrow and the gut are rich in soluble cues and structural proteins organized into complex cell microenvironments. However, this is an oversimplification of the highly developed signaling cascade that is orchestrated by the ECM. As our understanding of matrix and biology increases, new developments have revealed the dynamic nature of ECM and its role in tissue regeneration, aging,^[^ ^4^, ^5^ ^]^ disease progression,^[^ ^6^, ^7^ ^]^ and immune response.^[^ ^8^ ^]^

Given the capacity of the ECM to regulate cell fate, tissue engineering (TE) strategies aim to leverage its potential to provide a biomimetic platform and instruct cell behavior. Conventional TE approaches deliver cells using biomaterial scaffolds and external cues that mimic their native microenvironment. These scaffolds are often composed of ceramics, metals, or polymers, and they are designed to achieve a certain level of tissue induction through the inclusion of specific architectures, functional cues, or growth factors. As a biomaterial, ECM can be considered a collection of proteinaceous cell‐derived materials. ECM can not only be used as a scaffold but also as a supporting carrier for relevant bioactive compounds. For some applications, the role of ECM in cell modulation is to instruct cell fate. For example, the high presence of collagens can upregulate matrix metalloproteinase secretion, which is critical to orchestrating wound healing.^[^ ^9^ ^]^ The byproducts of ECM degradation play a key role in modulating macrophage phenotype, although this mechanism is poorly understood.^[^ ^10^ ^]^

**Figure 1:**
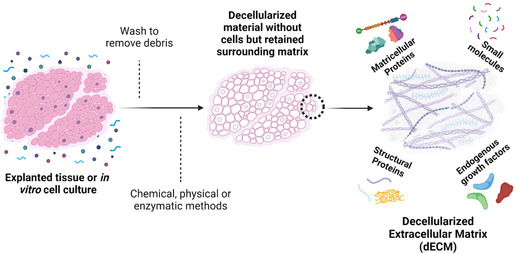
Decellularization of the ECM preserves its structure and composition. Depending on the method of decellularization, the result is a complex biomaterial that has significant advantages in the field of regenerative medicine due to its natural combination of structural proteins, small molecules, and growth factors.

The use of ECM‐based TE constructs offers cells their preferred microenvironment and can present substrate‐associated morphogens to potentiate tissue formation. However, several challenges hinder the use of ECM as a clinically relevant biomaterial including source variability, low production yields, and immune reaction.^[^ ^11^ ^]^ Herein, we will describe the main components of ECM and their relation to its bioactive potential, current strategies to remove cellular components within the ECM (decellularization), and methods to preserve and improve ECM bioactivity within TE constructs (**Figure** **1**). We will emphasize the use of decellularized ECM (dECM) for osteogenic, chondrogenic, and vascularization TE approaches. We will also describe current trends, necessary developments, and challenges in the use of dECM as a biomaterial substrate for clinical applications.

## 2 ECM Composition

The backbone of the ECM matrisome includes structural proteins such as collagens, proteoglycans, glycoproteins, and elastin. ≈300 proteins have been identified as core ECM components present in most tissues, and collagens represent the most abundant component. Different collagen subtypes are expressed according to the tissue and cell microenvironment that provide mechanical support, facilitate cell adhesion, control cell organization, and define tissue architecture. The clear abundance of collagen in the cell environment led to the first attempts of ECM‐inspired biomaterials that used collagen (recombinant, animal, or human‐sourced) for cell delivery and tissue regeneration.^[^ ^12^ ^]^

Collagen‐based technologies were developed to harness its biochemical and structural signatures that are imbued by the arrangement of proline‐lysine‐hydroxyproline residues. One of the earliest milestones in collagen‐based scaffolds was the introduction of the artificial skin membrane Integra, a bilayer construct composed of collagen, chondroitin sulfate, and silicone.^[^ ^13^ ^]^ The onset of medical‐grade collagen invigorated the development of collagen‐based strategies and products. However, poor mechanical properties of collagen‐based products limited their applications in musculoskeletal regeneration, leading to the need for composite scaffolds or combination with other materials. Collagen has also been chemically modified to change its biophysical characteristics. For example, compared to telocollagen that retains the short amino acid sequences at the end of the collagen molecules (telopeptides), atelocollagen is prepared by removing non‐helical telopeptides using enzymatic methods. These structural changes result in reduced host immune response, enhanced bioavailability, and improved mechanical properties, making atelocollagen a more attractive biomaterial.^[^ ^14^ ^]^

Further developments led to ECM‐inspired derivatives such as gelatin and hyaluronic acid that provide cells with microenvironmental cues that resemble those of the ECM.^[^ ^15^ ^]^ Gelatin is derived from collagen denaturation due to a series of post‐processing steps, conventionally acidic hydrolysis. Gelatin preserves many of collagen's biochemical properties with the caveat of losing the prototypical helix structure of collagen that enables its fibrillar structure. Gelatin is a clear example of the importance of preserving structural as well as chemical characteristics of the ECM components, as studies have shown differences in cell response between collagen‐ and gelatin‐based scaffolds.^[^ ^16^ ^]^ Furthermore, modification of gelatin with methacryloyl results in a photocrosslinkable polymer that, when combined with a photoinitiator and exposed to UV light, yields a hydrogel that is highly cell compatible and widely used for bioprinting.^[^ ^17^, ^18^ ^]^

Proteoglycans, proteins that have a glycosaminoglycan (GAG) side chain attached, represent the second most abundant component of ECM. There are four types of GAGs: hyaluronic acid, keratan sulfate; chondroitin/dermatan sulfate; and heparan sulfate, all of which are sulfated except for hyaluronic acid. The negatively charged GAGs enable the proteoglycans to sequester water and divalent cations, conferring space‐filling and lubrication functions. Moreover, GAGs facilitate the binding and presentation of growth factors and receptors to neighboring cells, thereby imbuing additional bioactivity to the ECM.^[^ ^19^ ^]^ The rich concentration of GAGs and proteoglycans make ECM‐derived platforms versatile drug carriers that can sequester, protect, and deliver therapeutic compounds with less risk of systemic side effects due to effective drug‐ECM absorption and control release.^[^ ^20^ ^]^ The readers are directed to a recent review that highlights new developments in the use of dECM‐based and ECM‐mimetic platforms for drug delivery.^[^ ^21^ ^]^

Single protein‐based constructs are unable to influence cell behavior in the same manner as ECM scaffolds, emphasizing the importance of protein interplay and the complexity of the ECM. Beyond its main structural proteins, the ECM includes actin filaments, intermediate filaments such as keratins or vimentin, ECM‐degrading enzyme motifs, matrix‐bound extracellular vesicles (EVs), and soluble cues such as growth factors that enhance cell proliferation and differentiation.^[^ ^22^, ^23^, ^24^ ^]^ The ECM's complex and dynamic environment is challenging to replicate in vitro using biomaterials formed from individual ECM components further motivating the importance of dECM.

**Figure 2:**
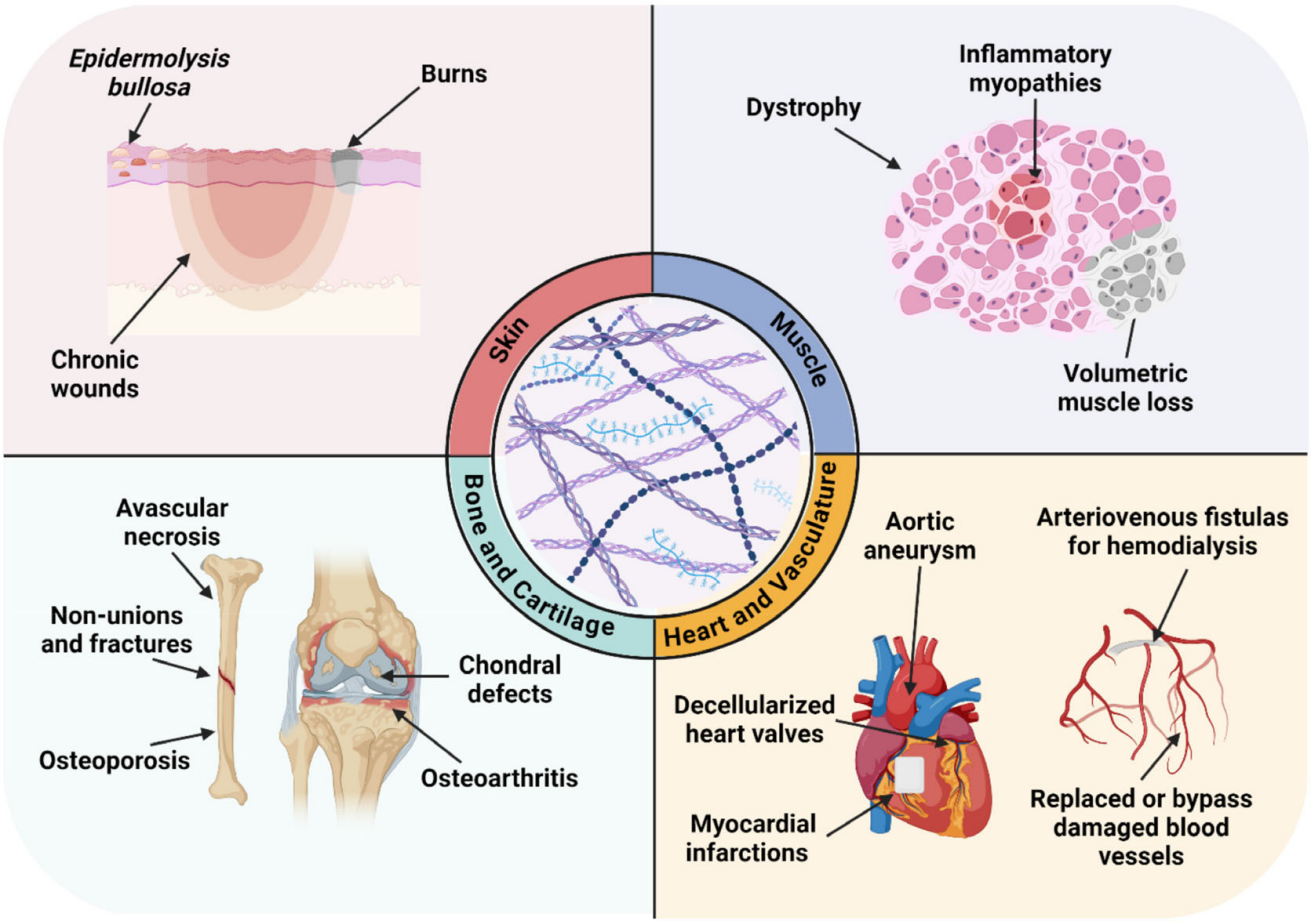
Decellularized ECM (dECM) can be employed as a potent biomaterial for several clinical applications. Summary of current and potential tissue or cell‐derived dECM approaches used to treat a plethora of conditions and diseases in bone, cartilage, skin, muscle, and cardiovascular tissues.

Although ECM clearly plays a key role in disease development, the precise mechanisms and relation to its composition remain elusive.^[^ ^25^ ^]^ In general, most ECM‐related disorders are caused by decreases in protein expression or changes in protein quality. For example, the Ehlers–Danlos family of syndromes is characterized by the changes in synthesis and processing of several types of collagens that lead to fragile connective tissue, hyperextensible skin, and hypermobile joints.^[^ ^26^ ^]^ Dystrophic epidermolysis bullosa (DEB), a group of genetic blistering disorders, is derived from mutations in the type VII collagen gene, encoding a large collagenous protein that is the primary component of anchoring fibrils at the dermal‐epidermal junction.^[^ ^27^ ^]^ Systemic conditions such as diabetes also affect ECM composition and remodeling. Diabetes alters collagen composition through the formation of advanced glycation end products (AGEs) associated with high glucose levels, decreased production of elastin and GAGs, and impaired ECM degradation and deposition due to aberrant matrix metalloproteinase (MMP) activity.^[^ ^28^ ^]^ In the case of musculoskeletal disorders, conditions such as osteogenesis imperfecta and Duchenne or Becker muscular dystrophies are caused by upregulation, genetic deletions, or disruption of ECM‐related proteins.^[^ ^29^ ^]^ The examination of ECM derived from patients with such disorders may provide an opportunity for creating innovative 3D models to study disease. Craniosynostosis is a disease occurring in young children characterized by premature suture fusion of the skull. While specific genes have been implicated in the disease, differences in protein binding and protein–ECM interactions of osteoblast‐derived ECM have been reported when comparing healthy to craniosynostotic osteoblasts. Specifically, key bone‐forming morphogens such as bone morphogenetic protein 2 (BMP‐2) and transforming growth factor beta (TGF‐β)^[^ ^30^ ^]^ exhibit altered binding and release kinetics, suggesting that co‐localization and sequestration within the ECM may contribute to the rate of osteoblast difference and suture fusion. However, it is crucial for any ECM‐based strategy that decellularization or processing steps do not alter the material to resemble the dysregulated environment of ECM‐related disorders, as this may lead to undesired cell behavior and hinder the regenerative potential of TE constructs. **Figure** **2** shows a summary of conditions and diseases that are currently being treated using dECM‐based products.

## 3 Generating ECM for Decellularization and Application as a Biomaterial Substrate

### 3.1 Cell Sourcing

We and others have harnessed the capacity of cells in culture to deposit a cell‐secreted endogenous ECM for use as a biomaterial. The composition and bioactivity of the ECM are primarily bestowed by the cell source and culture conditions. However, the bioactivity of the ECM can be readily tuned to dictate its functional properties because cells modify their secreted ECM products in response to various stimuli, including mechanical cues, cell density, oxygen and nutrient concentration, and other factors that constitute the microenvironmental niche.^[^ ^31^ ^]^

ECMs can be derived from various cell populations, combined from multiple sources for use in one construct, or generated from a co‐culture system to yield specific compositions or cellular responses. Indeed, ECM secreted from bone marrow‐derived mesenchymal stromal cells (BMSCs), adipose stromal cells (ASCs), and dermal fibroblasts had distinct compositions but supported comparable cell adhesion and survival while inducing similar osteogenic differentiation of ASCs in culture.^[^ ^32^ ^]^ ECM secreted by fetal MSCs accelerated the expansion of adult MSCs in culture, demonstrating the influence of age on the bioactivity of cell‐secreted ECM.^[^ ^33^ ^]^ Moreover, one can potentiate the regenerative capabilities of a cell construct in vitro by introducing distinctive ECMs unrelated to the final tissue structure or composition,^[^ ^34^ ^]^ enabling a plethora of combinations and compositions for ECM‐based scaffolds.

MSCs are widely known for their role in regeneration and homeostasis across several tissues. Their rapid expansion in vitro and potential to differentiate into cells comprising tissues of the musculoskeletal system make them promising candidates to generate ECM for a variety of clinical applications. The first characterization studies of MSC‐derived ECM examined its composition and effect on chondrogenic differentiation.^[^ ^35^ ^]^ These findings demonstrated the importance of biomolecule deficiency, availability of soluble cues, and protein upregulation within the MSC microenvironment. Subsequent studies established the potential of enriching the GAG content of MSC‐secreted ECM for growth factor retention^[^ ^32^, ^36^ ^]^ and the capacity to control ECM composition by altering culture conditions such as initial cell seeding density, culture duration, and oxygen tension.^[^ ^31^ ^]^

Inspired by tissue heterogeneity, heterotypic co‐culture systems are under investigation to increase bioactivity of the ECM compared to homotypic culture approaches. ECM secreted by BMSCs cultured with human umbilical vein endothelial cells (HUVECs) had a higher fibronectin and laminin content compared to controls and constructs seeded with BMSCs. Furthermore, this study showed the potential of cell‐secreted ECM to upregulate osteogenic genes in an MSC‐HUVEC co‐culture system.^[^ ^37^ ^]^ Nonetheless, there is a gap in knowledge regarding whether these effects could be achieved by introducing a mixture of ECMs upon construct fabrication or if this is the result of cell interactions and soluble cues during cell culture.

A key challenge of employing cell‐secreted ECM as a biomaterial relates to low cell proliferation and donor variability of the cell source. The discovery of induced pluripotent stem cells (iPSCs) provided an alternative to this limitation by allowing the indefinite expansion of iPSCs that can be further differentiated into any cell type.^[^ ^38^ ^]^ However, iPSC‐derived populations are unique cell types compared to their somatic counterparts, and more studies are needed to understand differences in the ECM produced by iPSC‐derived cells. Moreover, the reprogramming of somatic cells had a “rejuvenation” effect on cell function. Compared to somatic MSCs, reprogrammed iPSC‐MSCs obtained from the same cell population exhibited a decrease in tissue‐specific and replicative senescence, DNA methylation, increased proliferation capacity, and improved trilineage differentiation potential.^[^ ^39^, ^40^ ^]^ This phenotypic change would, in theory, alter the composition of the ECM produced by reprogrammed progenitor cells, although no studies have reported differences between somatic and reprogrammed MSC‐secreted ECM to date. Nonetheless, iPSC‐MSC‐derived ECM can potently instruct osteogenic and chondrogenic potential of human BMSCs,^[^ ^41^ ^]^ bringing a new perspective on the use of cell‐secreted ECM to harness the superior proliferative nature of iPSCs.

Although unconventional, plant‐derived ECM has been explored for highly specific vascularization approaches, mostly due to its interconnected nature, hydrophilicity, and mechanical properties.^[^ ^42^ ^]^ Studies with plant ECM highlight the importance of composition and cellulose microstructure for controlling cell proliferation and orientation, respectively. However, most of these studies are exploratory and have not yet reported benefits or improved clinical translation compared to animal‐derived ECM constructs and thus will not be further described in this review.^[^ ^43^ ^]^

### 3.2 Interplay Between ECM and Cellular Senescence

In recent years, our improved understanding of cellular senescence has revealed mechanisms on which healthy ECM composition can induce or protect from the characteristic senescent pro‐inflammatory environment.^[^ ^44^, ^45^ ^]^ Disturbances in cell–ECM adhesion can make cells more susceptible to chemotherapy and ionizing radiation, which are common triggers of cellular senescence.^[^ ^46^ ^]^ Studying the composition of skeletal muscle fibroblast ECM revealed differences in collagen content, fat infiltration (myosteatosis), stiffness, fiber orientation, and tortuosity between old and young individuals.^[^ ^47^ ^]^ These alterations correlated with increased expression of fibrogenic markers and decreased myogenicity for old fibroblasts.^[^ ^48^ ^]^ The current hypothesis behind these changes is the abnormal autocrine and paracrine activity of senescent cells known as the senescent‐associated secretory phenotype (SASP).^[^ ^49^ ^]^

Although the systemic effect of the SASP is poorly understood, it is theorized that dysregulation of MMPs and tissue inhibitor metalloproteinases (TIMPS) has a detrimental impact on ECM production and cell‐ECM communication that can lead to age‐related fibrosis, atherosclerosis, and articular cartilage degeneration.^[^ ^4^, ^50^, ^51^ ^]^ This dysregulated remodeling is regulated by the abnormal expression of specific structural proteins in the ECM such as collagens II and III, laminin, and TGF‐β,^[^ ^52^, ^53^ ^]^ and highlights the intrinsic relationship between ECM expression and remodeling. Interestingly, dECM from cells derived from young individuals, when used as a substrate for the culture of cells from older donors, has a rejuvenation effect and can restore proliferative capacity and differentiation potential.^[^ ^33^, ^54^ ^]^ In another example, human umbilical cord‐derived MSCs cultured on their own dECM exhibited an attenuated response to reactive oxygen species (ROS)‐induced senescence, which is mediated by the silent information regulator type I (SIRT1) signaling pathway and collagen I interactions.^[^ ^55^ ^]^ This phenomenon emphasizes the importance of ECM–cell interactions, ECM bioactivity, and its potential to instruct cell behavior for therapeutic applications.

### 3.3 Production of Decellularized ECM

#### 3.3.1 ECM Deposition

ECM deposition, as well as composition, is influenced by external stimuli such as substrate stiffness, addition of soluble factors, and mechanical stimulation such as fluid flow. The rate of ECM deposition is dependent upon cell source, with low passage cells often preferred due to relative differences in cell proliferation and to avoid producing ECM with an aberrant protein composition that is characteristic of senescent cells.^[^ ^56^ ^]^ A similar trend in protein dysregulation has also been reported between cells from younger versus older donors when keeping the passage number equal.^[^ ^57^ ^]^ Acknowledging that most investigations culture cells on tissue culture plastic, the introduction of soluble cues to cell culture media is a facile approach to alter the composition or increase ECM production. The supplementation of culture media with L‐ascorbic acid‐2‐phosphate (A2P) increases ECM deposition, GAG content, and collagen production for many cell types.^[^ ^58^ ^]^ Macromolecular crowding (MMC) is another strategy to accelerate and enhance the deposition of ECM.^[^ ^59^ ^]^ This technique creates an excluded‐volume effect that affects the conformational freedom of the system, leading to elevated basal free energy of the reactant macromolecules. The thermodynamic change alters protein folding, creates complexes with longer half‐lives, and produces a resilient environment that protects from pH, temperature, and ionic strength changes.^[^ ^60^ ^]^ Importantly, the use of MMC agents increases GAG content within cell‐secreted ECMs, resulting in improved retention of endogenous heparan sulfate‐binding growth factors. In one example, ECM secreted by MSCs in the presence of MMC agents was functionalized by adsorption of exogenous BMP‐2.^[^ ^36^ ^]^ Nonetheless, current strategies for dECM production are limited by variability in composition and a poor understanding of the relation between batch‐to‐batch differences and bioactive potential.

Mechanical properties and 3D architecture can also instruct cell behavior by controlling spreading, contractility, and migration.^[^ ^61^ ^]^ Mediated through mechanoreceptors such as integrins, TRPV4, and PIEZO1, the capacity of cells to sense the stiffness of their underlying substrate regulates cell phenotype, which has a subsequent effect on the composition, bioactivity, and stiffness of the secreted ECM. In fact, tumorigenesis and tumor progression are believed to be intrinsically related to ECM stiffness.^[^ ^62^ ^]^ Substrate stiffness can alter ECM deposition and resultant composition. Early studies of the interplay of integrins in mechanosensing and the effect of stiff substrates, such as tissue culture plastic in 2D cell culture, report changes in ECM protein expression and remodeling in response to mechanical cues.^[^ ^63^ ^]^ However, the relation between compliant and stiff substrates is cell‐type specific and requires a deep understanding of cell mechanobiology and tissue development. Substrate stiffness is a potent cue that influences lineage‐specific commitment of MSCs by altering the expression of non‐collagenous ECM components and soluble cues.^[^ ^64^ ^]^ This behavior has also been observed in endothelial cell culture in ECM‐inspired substrates with a range of stiffness, influencing the expression of angiogenesis‐related genes and formation of capillary networks.^[^ ^65^ ^]^ The interaction of dermal fibroblasts with compliant substrates decreases TGF‐β activation, downregulates collagen I and II gene expression, increases cell motility, and decreases cell size.^[^ ^66^, ^67^ ^]^ In contrast, substrates with high stiffness promote osteogenesis in MSCs, representing effective tools to promote tissue mineralization.^[^ ^68^ ^]^ Compliant substrates are better for preserving MSC phenotype and can improve differentiation toward the adipogenic and chondrogenic lineage.

Beyond the passive mechanical stimulation represented by substrate stiffness, active mechanical stimulation represents another approach to induce and influence ECM deposition by stimulated cells and drive cell differentiation.^[^ ^69^ ^]^ Daily mechanical stimulation improved MSC‐secreted ECM quality and alignment.^[^ ^70^ ^]^ The interplay of both chemical and mechanical stimulation has been explored to improve the quality and quantity of a TE tendon construct consisting of MSCs and HUVECs.^[^ ^71^ ^]^ Cyclical stretching and tenocytic extract (intracellular proteins and genetic material of lysed chondrocytes) significantly increased tendon‐related gene expression. The relevance of mechanical stimuli during ECM production was also reported when bone cells were exposed to shear stress similar to what occurs during mechanical loading of bone through fluid flow inside the canalicular–lacunar and trabecular spaces within bone tissue.^[^ ^72^ ^]^ However, changes in the bioactivity of dECM scaffolds due to substrate stiffness during ECM deposition have yet to be reported and may reveal another approach to control cell function during ECM production. Mechanical regimes often fail to recapitulate the dynamic forces at which tissues are commonly exposed, leaving room for the development of complex bioreactors that can apply different frequencies, amplitudes, and mechanical stimuli (compression, shear stress, or stretching).

Three‐dimensional culture systems are under investigation to more accurately mimic the in vivo matrix architecture. For example, osteoconductive dECM was generated by seeding cells in a fibrous scaffold that was subsequently decellularized.^[^ ^73^ ^]^ Structural cues may be used to guide cell deposition, which is particularly desirable for highly organized tissues.^[^ ^74^ ^]^ Furthermore, combining a 3D culture environment with the appropriate mechanical stimulation yields an ECM with similar composition to native tissue and offers advantages in total matrix deposition, cell proliferation, differentiation, and mechanical stability. Additionally, the creation of substrates with nanopatterns for use as cell culture substrates enables the study of topographical effects on ECM deposition. For example, dECM deposited in a nanogrooved surface increased MSC vinculin production, upregulated COL1A1, COL2A1, and ACAN gene expression and promoted chondrogenic differentiation while suppressing osteogenic differentiation.^[^ ^75^ ^]^ Scaffold‐free approaches may also be used to produce dECM with improved bioactive properties. The use of cell aggregates (spheroids) has garnered momentum in the development of new TE strategies due to their capacity to better recapitulate cell–cell and cell–ECM interactions. Spheroid‐derived dECM produced using MMC approaches exhibited superior growth factor retention and vascularization capabilities both in vitro in a tube formation assay and in vivo.^[^ ^76^ ^]^ Other strategies for increasing ECM production include the addition of essential enzymes related to collagen production, growth factors such as TGF‐β and fibroblast growth factor (FGF), low serum concentrations, changes in oxygen tension, and gene‐editing technologies. Nonetheless, scalability and consistency remain major challenges in ECM production due to the high costs associated with large‐volume bioreactors, which becomes more apparent for ECM produced from complex co‐culture systems.^[^ ^77^ ^]^

**Figure 3:**
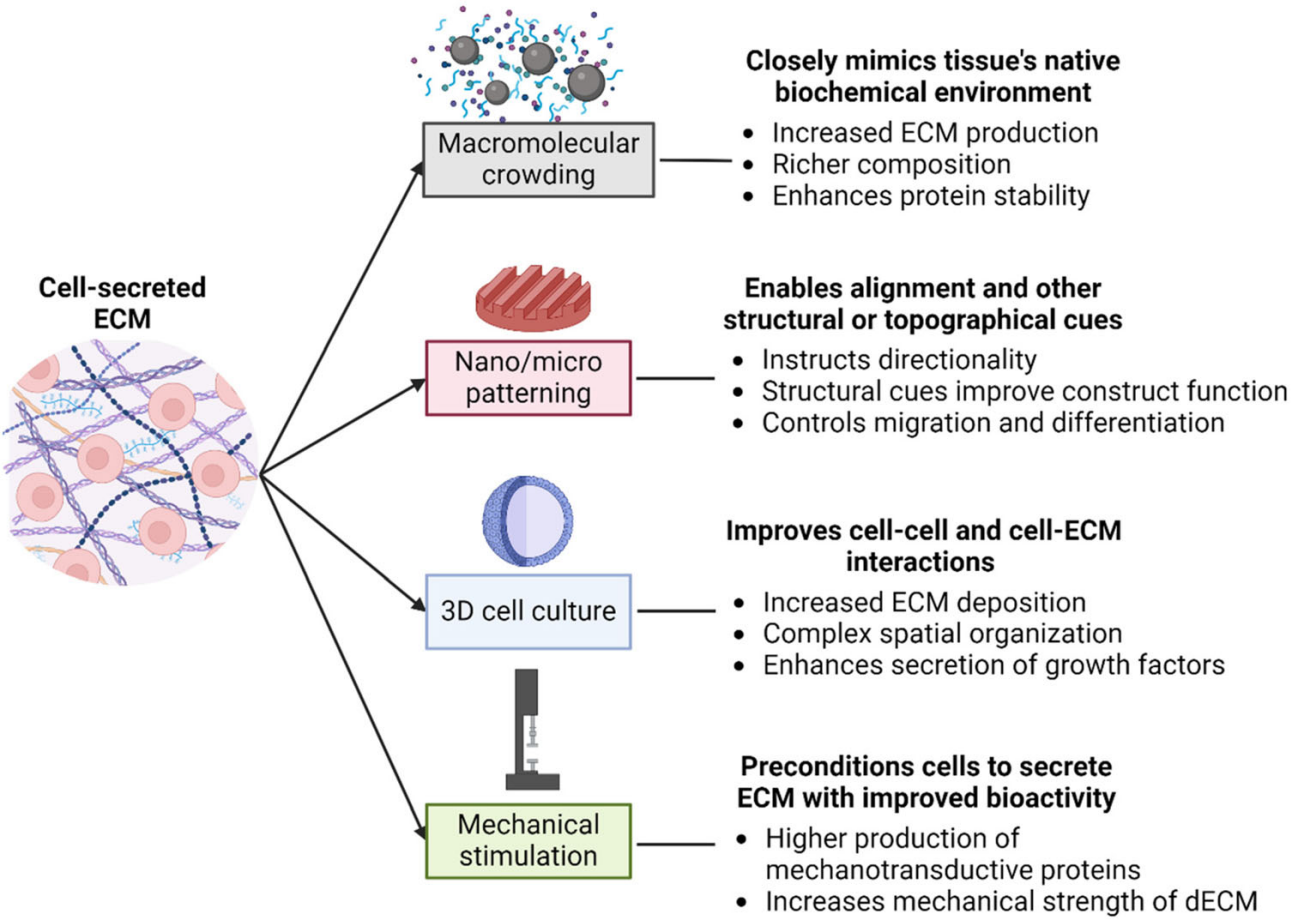
Physical and biochemical stimuli can increase dECM deposition and alter its bioactive properties. Schematic of common strategies used to improve or enhance the deposition of cell‐secreted dECM. Most approaches focus on a specific aspect of dECM functionality, although they often affect both composition and structure.

Recent developments in “click chemistry,” which are high‐yield and highly specific chemical reactions that proceed in biological environments without side reactions toward endogenous functional groups, facilitate metabolic labeling of ECM‐derived GAGs that can be tagged by bioorthogonal chemical reporters. The term “clickECM” refers to ECM produced from cells that used bioorthogonal functional groups as building blocks for proteins or glycans. After decellularization, the dECM may be further functionalized by the addition of a probe‐bearing functional group. This novel technology was implemented in the fabrication of fibroblast, MSC, and chondrocyte‐derived dECM and provided key information on matrix remodeling, cell–matrix interaction, and tissue regeneration.^[^ ^78^, ^79^ ^]^ Gene editing is another strategy for engineering ECMs to overcome specific challenges of pathological conditions, which is further discussed in the Future Outlook section of this review. **Figure** **3** provides an overview of common strategies to improve the bioactivity of cell‐secreted dECM.

#### 3.3.2 ECM Decellularization

The efficient removal of cellular components is critical for translation of all ECM‐based scaffolds. The standard for DNA removal from cell‐secreted and tissue‐derived dECM is less than 50 ng of DNA per mg of tissue.^[^ ^80^ ^]^ Achieving this benchmark is especially crucial for xenogeneic and allogeneic dECM scaffolds to avoid an immunogenic response.^[^ ^81^ ^]^ However, trace DNA is not solely responsible for the immunogenic properties of ECM. Damage‐associated molecular patterns (DAMPs) can mediate immune interaction with dECM constructs. Aside from nuclear and mitochondrial DNA and ROS, DAMPs signals also originate from fragmented byproducts of a harsh decellularization process.^[^ ^82^ ^]^ Furthermore, differences in innate immune response in vivo have been reported as a function of ECM source. For example, decellularized bone‐derived ECM resulted in higher recruitment of monocytes than cardiac ECM, emphasizing the necessity for further characterization.^[^ ^83^ ^]^ These adverse effects of dECM highlight the need for a deep understanding of the relationship between ECM composition and structure of the cell microenvironment for proper tissue function.

The effectiveness of the decellularization method is dictated not only by residual DNA content but also changes in bioactivity due to the removal process. For example, most DNA removal processes rely on harsh detergents (e.g., Triton X‐100 or sodium dodecyl sulfate (SDS)) that strip the essential heparan sulfate proteoglycans from the substrate, thus diminishing the capacity of the dECM to interact with heparin‐binding growth factors.^[^ ^80^ ^]^ To overcome this limitation, novel physical methods have emerged as alternatives to conventional detergent‐based methods, while chemical methods utilize enzymatic removal or targeted degradation of specific cellular components.^[^ ^84^ ^]^ **Table** **1** describes the most common decellularization methods and their overall efficiency as it relates to DNA removal and preserving bioactivity. Importantly, a majority of these techniques were developed for the decellularization of whole explanted tissues and required convective transport to perfuse tissues with these reagents. Decellularization of in vitro cell‐secreted ECM has fewer limitations in terms of diffusion energy and mass transfer, and thus may be achieved more rapidly and with gentler methods. Moreover, the lack of macro‐architecture in cell‐secreted ECM permitted the development of decellularization methods that prioritize composition by employing physical methods that would otherwise compromise key structures present in tissue‐derived ECM. Nonetheless, perfusion technologies represent a key alternative to solve the challenges with diffusion in tissue decellularization.^[^ ^85^ ^]^ This process allows for penetration and improved removal of cellular components and has been successfully used to create scaffolds from decellularized tissues, including lungs, heart, liver, and kidneys.^[^ ^86^, ^87^, ^88^, ^89^ ^]^

**Table 1:** Conventional decellularization methods used to produce dECM are grouped by their main processing mechanism.

Mechanism	Decellularization method	Mechanism and known effects	References
Physical	Thermal shock (freeze/thaw)	Preservation of protein content and mechanical properties without the need for detergents. This can lead to degradation due to the formation of water crystals that damage the ECM structure.	[184]
	Electroporation	Disruption of cell membrane by the application of electric fields. The non‐thermal mechanism preserves structure by introducing nanopores into the lipid bilayer.	[185]
	Ultrasonic	Uses cavitation to mechanically disrupt the cells. Ultrasonic waves are often alternated with washes of low‐concentration detergents. Enables improved diffusion but requires extensive optimization.	[92, 186]
	Supercritical fluid	Carbon dioxide supercritical fluid facilitates non‐toxic removal of cellular components. Proven sterilization method that preserves bioactivity in biological materials.	[187]
	High hydrostatic pressure	Hydrostatic pressurization to remove cells, bacteria, and viruses. Subsequent washes are required to remove residual cellular components. Highly efficient DNA removal and no further sterilization required.	[188]
Chemical	Surfactants/Detergents	Amphipathic compounds dissolve non‐polar components. Detrimental for ECM composition and requires additional wash steps for safe detergent removal. Inexpensive and highly scalable.	[189]
	Alcohols	Disrupts cell membrane by dehydration, solubilizes lipids, and serves as an antimicrobial agent. However, this approach can disrupt ECM structure by inducing undesired collagen crosslinking.	[190]
	Acids and bases	Acids and bases are used to dissociate DNA and denature ECM proteins. Organic acids are commonly used but can induce damage and removal of collagen.	[184, 191]
	Enzymatic	Nucleases (DNAse or RNAse), proteases (trypsin or collagenase), and lipases are applied to decellularize. This technique enables removal of specific cellular components and additional functionalization.	[192]

Preserving the composition and 3D structure of the ECM is critical when decellularizing either highly organized tissues or cell‐secreted matrices. Recently, the combination of decellularization methods has proven to be the most efficient way to remove cellular components without compromising ECM bioactivity and structure. TE scaffolds have been prepared by combining physical decellularization methods such as electroporation and sonication^[^ ^90^ ^]^ or chemical methods such as the use of detergents and DNA enzymatic degradation.^[^ ^91^ ^]^ Other novel strategies involve the use of a primary physical decellularization method followed by a mild chemical step.^[^ ^92^ ^]^ This approach harnesses the capacity of physical methods to “loosen” the ECM and remove most of the cellular components, followed by a lower concentration or shorter chemical method, normally enzymatic, to complete the removal of DNA. Recently, a new branch of decellularization methods that uses reactive oxygen or nitrogen species has proven effective in the removal of cellular components while also enhancing antioxidant response and altering macrophage polarization toward an anti‐inflammatory phenotype.^[^ ^93^ ^]^ This paradigm shift could inspire the development of other multifunctional decellularization protocols in the future.

#### 3.3.3 ECM Functionalization

Given the high‐bioactive potential of ECM, there is a paucity of examples of ECM functionalization with additional biochemical cues when applied to TE constructs. However, depending on the application, ECM may be further functionalized to enhance its regenerative potential. Covalent functionalization is usually avoided, as chemical byproducts or uncontrolled protein crosslinks can hinder ECM function. Nonetheless, custom‐made peptides with cell adhesive sequences such as RGD and REDV have been successfully used to functionalize ECM for endothelial cell adhesion and migration.^[^ ^94^ ^]^ Additionally, copper‐catalyzed click chemistry facilitated the integration of biomimetic peptides without compromising ECM composition.^[^ ^95^ ^]^ Because of their synthetic nature, these tunable peptides can be designed to degrade under specific cell activity, have less batch‐to‐batch variability, and enable the decoupling of stiffness from bioactivity, unlike conventional dECM approaches. Recently, such synthetic peptides were coupled to cell‐secreted dECM to mimic VEGF and enhance angiogenesis.^[^ ^95^ ^]^ However, low bioavailability, poor stability, and risk of eliciting immune response represent current major limitations of introducing synthetic peptides into TE constructs.^[^ ^96^ ^]^

In contrast to chemical modifications, physical entrapment of materials via ECM immobilization or gelation is employed to load and deliver bioactive compounds in a controlled manner. For example, the inclusion of osteogenic granules,^[^ ^97^ ^]^ poly(lactic‐co‐glycolic acid) (PLGA) microspheres as carriers of small molecules,^[^ ^98^, ^99^ ^]^ and conductive nanoparticles^[^ ^100^ ^]^ have all been pursued to increase tissue mineralization, instruct tissue regeneration, and improve cell differentiation and maturation. Because functionalization by physical methods uses ECM as a carrier, chemical modifications are not required to control the mechanical properties of the ECM construct.

Although it is possible to modify ECM degradability via chemical crosslinking, research suggests the regenerative potential is connected to the early presence of byproducts of ECM remodeling, and delays in their degradation may lead to scar tissue formation.^[^ ^101^, ^102^ ^]^ Common strategies to slow the degradation rate of dECM are the inclusion of crosslinking agents such as glutaraldehyde, genipin, carbodiimides, induction of endogenous collagen crosslinks using enzymes,^[^ ^103^ ^]^ or photo‐activated crosslinking agents, while collagenase or trypsin are used to accelerate dECM degradation and adsorption.^[^ ^104^, ^105^ ^]^ The incorporation of functional groups that are degraded upon exposure to chemical or physical environmental cues represents an innovative frontier that may yield biomaterials responsive to changes in disease state or the microenvironment.

### 3.4 Characterization of Decellularized ECM

The composition of dECM, influenced by culture variables and the impact of the decellularization process, is a key attribute that imbues its biological properties. The complexity of dECM requires a thorough characterization of its chemical, physical, and structural properties to assess its potential for tissue regeneration. Common assays for ECM chemical characterization include colorimetric assays for major biomolecules such as protein concentrations, 1,9‐dimethylmethylene blue (DMMB) assays for sulfated GAGs, and 4‐(dimethylamino)benzaldehyde (DMAB) assays for collagen content. SDS‐PAGE, Western Blot, immunohistochemical analysis, infrared spectroscopy, high‐performance liquid chromatography (HPLC), and proteomic analysis (N‐terminal amino acid sequencing and mass spectrometry) are other common methods employed to identify and quantify specific biochemical components of the dECM. The structural and physical characterization of the dECM, including fiber diameter, collagen subtype, and collagen crosslinks can be interrogated using high‐resolution microscopy techniques (e.g., scanning electron microscopy, second harmonic generation imaging, or time‐resolved fluorescence spectroscopy).^[^ ^103^ ^]^ Finally, mechanical characterization using atomic force microscopy or tensile testing could indicate changes in composition, collagen crosslinking, and chemical structure due to decellularization.^[^ ^106^, ^107^ ^]^ Recent studies used traction force microscopy to analyze cell–ECM interaction and understand the relationship between substrate stiffness and mechanotransduction.^[^ ^108^ ^]^

**Figure 4:**
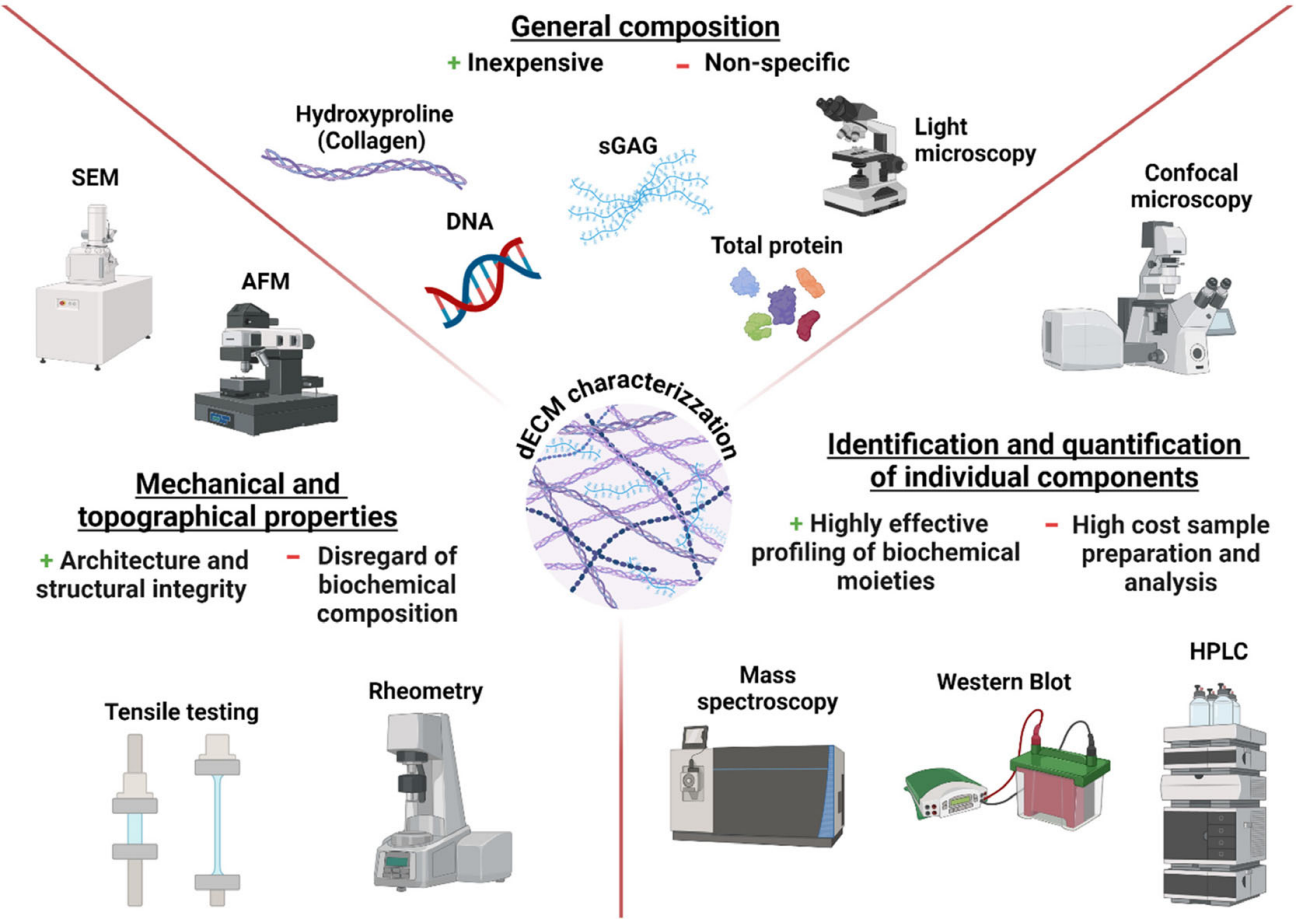
Comprehensive characterization of the bioactive potential of dECM requires a deep understanding of its physical and biochemical properties. Summary of common techniques for dECM characterization, their main advantage and limitation. The selection of techniques from different categories is necessary to fully study the effects of dECM on tissue regeneration.

The most critical challenge for ECM characterization as it relates to clinical translation is to rapidly assess lot‐to‐lot variability. Most characterization methods require time‐consuming sample preparation and substantial resources to ensure quantity, composition, and consistency. Current techniques to quantitatively assess protein concentration and purity for protein‐based medical products are HPLC, Kjeldahl and Bradford methods, and bicinchoninic acid (BCA) assays. These protocols are insufficient to assess the reproducibility of dECM and do not account for differences in GAG content that influence bioactivity. **Figure** **4** summarizes the most common techniques for dECM characterization.

Beyond accurately quantifying composition, the use of dECM‐based products requires evidence of sufficient sterilization before clinical use. Current international standards set by the US Food and Drug Administration (ISO 22442‐1 and ISO 13408‐1) stipulate that animal dECM‐based constructs should undergo terminal sterilization to remove any harmful components that could compromise function or cause infection.^[^ ^109^ ^]^ FDA guidelines do not require a terminal sterilization method for allogeneic dECMs. Nonetheless, conventional sterilization methods used for medical devices, such as autoclaving, ethylene oxide, or ionizing radiation, are inadequate for dECM and can denature the protein components. Supercritical carbon dioxide is the most accepted sterilization method for dECM products, as it causes minimal damage or changes to structure, mechanical properties, and bioactivity.^[^ ^110^ ^]^ Other sterilization alternatives include peracetic acid and ultraviolet irradiation, although their effects on dECM composition and mechanical strength remain unexplored.

## 4 ECM‐Based Tissue Engineering Strategies

The principles of modern tissue engineering can be traced to early attempts to use processed tissues (such as bone or skin) as scaffolds that support cell growth and promote tissue regeneration. Since the 1970s, the use of individual organic and inorganic components of tissues to support tissue formation established the importance of the ECM as a platform for tissue formation in vitro or ex vivo. As TE strategies continued to evolve, new materials were developed to mimic the native composition and architecture of the ECM. Most approaches focused on mimicking one or two components of the ECM or recapitulating the micro‐ or even nanostructure of native tissue. However, new TE strategies recognize the need to reintroduce the dynamic and hierarchical nature of the tissue ECM. Thus, the use of ECM secreted by relevant cell types offers the complexity of the ECM network without the reliance on decellularizing whole tissues or the challenges associated with functionalizing synthetic polymers.

Recellularization of decellularized tissues gained momentum during the 2000s when native cell populations re‐introduced into decellularized scaffolds promoted at least partial recovery of function.^[^ ^86^, ^87^, ^88^ ^]^ However, extended viability and continuous function of recellularized tissue remained elusive, and early attempts at recellularizing failed primarily due to a harsh decellularization process that compromised the biochemical and mechanical integrity of the dECM.^[^ ^111^ ^]^ Decellularization strategies have drastically improved in the last decade with the introduction of effective perfusion systems, mild decellularization methods (physical and biochemical), and protocols designed for specific tissue structures. These advancements have led to recellularized tissues that exhibit prolonged viability, increased recovery of function, and are showing promise as potential alternatives for full organ transplantation.

The onset of technologies that facilitate the production and manipulation of ECM enabled a surge of scaffold‐free approaches. The most common of these techniques are cell bioprinting and cell sheet technology, both of which require the implementation of biomaterials as bioinks or vehicles to support associated cells.^[^ ^112^ ^]^ Cell differentiation and proliferation may be increased by the addition of dECM together with another biomaterial which provides tunable mechanical properties. This approach combines the benefits of the superior bioactivity of dECM with the tunable, wide‐ranging mechanical properties or degradation profile of the scaffolds, thus facilitating greater control over the biophysical properties of the construct.

### 4.1 Fabrication of ECM‐Based Constructs

After successful decellularization, dECM can be processed to achieve specific micro‐ or macrostructures. The biochemical signature of the ECM must be preserved by avoiding removal or addition of any moieties that can lead to undesired cell interactions. In contrast to synthetic polymers, the complexity of the ECM requires an understanding of the interplay of fabrication parameters on resultant composition and biophysical properties.^[^ ^113^ ^]^ For example, electrospun constructs require the application of an electric field to produce nano‐ or micron‐size fibers. For most linear synthetic and natural polymers, the selection of a non‐toxic solvent is a key challenge to preserve the chemical structure.^[^ ^114^ ^]^ However, endogenous growth factors and short peptides within dECM are more susceptible to organic solvents and high voltages that may compromise their bioactivity, requiring the selection of a solvent system with increased conductivity to optimize energy transfer. Micro or macro‐sized particles, sometimes referred to as micronized ECM, are commonly used to efficiently introduce dECM into scaffolds. This strategy has the advantage of using the architecture of the scaffolds instead of relying on modifications of the dECM. However, these constructs offer lower availability of the dECM moieties compared to bulk dECM approaches, thus limiting their bioactive potential.^[^ ^115^ ^]^

Hydrogels are versatile, tunable platforms that can be processed as scaffolds or injected directly at the injury site. Hydrogels enable the 3D culture of cells and control of construct geometry by manipulating the gelation parameters for crosslinking the hydrogel. The crosslinking approach is often chemical in nature, which depends on the selected material or any post‐processing modifications. However, because of the high collagen content in dECM, gelation kinetics can be controlled by a thermodynamic reversible process.^[^ ^116^ ^]^ Alternatives such as methacrylation for subsequent photocrosslinking or induction of chemical crosslinking by N‐hydroxysulfosuccinimide (NHS) have been successfully implemented for TE applications,^[^ ^117^ ^]^ although this approach may cause undesired protein crosslinking due to amine reactions that compromise the bioactivity of the ECM.^[^ ^118^ ^]^ Pepsin digestion is one strategy to solubilize the dECM components and then control gelation via a control acid‐base reaction.^[^ ^119^ ^]^ However, pepsin digestion decreases the bioactivity of the ECM due to broad‐spectrum protease activity that could digest growth factors entrapped in the material.

Another approach to fabricating ECM‐based hydrogels is combining dECM with other established biomaterials such as agarose, alginate, hyaluronic acid, and polyethylene glycol (PEG).^[^ ^115^, ^120^, ^121^, ^122^ ^]^ This approach enables control of microarchitecture and mechanical properties of the scaffold, yet the bioavailability of the dECM in such systems is dependent on the fabrication method and polymer‐dECM ratio. Alternatively, the inclusion of ECM‐inspired proteins within the hydrogel is an effective platform for maintenance and expansion of human cells.^[^ ^123^ ^]^ Enzymatic crosslinking has been employed to increase the mechanical properties and malleability of the dECM gel. Enzymes such as transglutaminase (meat glue) and lysyl oxidase (as a collagen crosslinker) have been used to increase mechanical stability without compromising dECM composition.^[^ ^124^, ^125^ ^]^

In contrast, scaffold‐free TE technologies focus on creating cell‐based constructs without the introduction of biomaterials. This category of TE constructs promotes cell–cell interaction through cell aggregation, cell sheets, and bioprinting to improve cell–cell and cell–ECM interactions, enhance tissue integration, and in the case of bioprinting, control the spatial distribution of cell populations and ECM. Several bioprinting efforts have sought to mimic the architecture of tissues and large organs.^[^ ^126^, ^127^ ^]^ Recently, the field has shifted to preserving construct function, perfusion of printed constructs, and generation of 3D constructs for individual patients. Nonetheless, a key challenge for bioprinting remains the development, selection, and fabrication of bioinks. Due to its ubiquitous presence within native tissues, collagen I remains one of the more common bioinks and has enabled the fabrication of a printable human heart.^[^ ^128^ ^]^ However, mechanical stability, thermal sensitivity, and relatively low printability are challenges in the translation of collagen constructs. dECM as a bioink was theorized since the conception of bioprinting, but poor printability and stability limited its use in TE. The first successful implementation of dECM bioinks was reported with a pH‐dependent pre‐gel method.^[^ ^129^ ^]^ Since then, new developments have improved bioink formulations by combining dECM with alginate,^[^ ^130^ ^]^ gelatin,^[^ ^121^ ^]^ gelatin methacrylate (GelMA),^[^ ^131^ ^]^ vitamin B2,^[^ ^132^ ^]^ or collagen.^[^ ^133^ ^]^

Extrusion‐based bioprinting is readily accessible and widely studied to build complex tissue structures. However, inkjet bioprinting offers advantages in printability when using low‐viscosity inks (<10 MPa) compared to conventional extrusion‐based technologies.^[^ ^134^ ^]^ Freeform reversible embedding of suspended hydrogels (FRESH) bioprinting improves spatial resolution and printability for low gelation inks such as dECM.^[^ ^128^ ^]^ In contrast, other developments such as laser‐assisted bioprinting have not been successfully implemented with dECM inks due to the risk of thermal degradation associated with laser radiation. As an alternative, concentrated cell suspensions have been used as bioinks or even the combination of bioink and cell aggregation techniques by bioprinting spheroids. The latter offers the advantages of spheroid platforms with the potential of using ECM‐derived bioinks to further instruct cell behavior.^[^ ^135^ ^]^ **Table** **2** summarizes commonly employed fabrication techniques to create dECM‐based constructs.

**Table 2:** Common fabrication technologies used to create dECM‐based constructs.

Type	Platform	Description	Ref
Scaffold‐based	Mineralized scaffolds	Composite scaffolds are fabricated by mixing mineral composites (hydroxyapatite) with ECM‐proteins (i.e., collagen or dECM particles) and biocompatible polymers (PLGA or PLA). The platform enables better control of mechanical properties and scaffold macro and micro architecture.	[115, 193]
	Electrospun constructs	Made from natural or synthetic polymers, these micro or nano fibers can be fabricated from dECM or coated with matricellular proteins. These can also be used to as culture substrates to generate ECM with specific architectures.	[194]
	Hydrogels	High water polymer network that closely mimics ECM microenvironment. Hydrogels can be fabricated from synthetic polymers or natural proteins (i.e., collagen, hyaluronic acid, alginate), followed by the inclusion of soluble dECM or microparticles. dECM gels are possible but have limited mechanical properties.	[195, 196]
Scaffold‐free	Cell aggregates	Self‐assembled cellular aggregates (spheroids) that mimic cell–cell interactions. Introducing cell‐secreted dECM or ECM‐inspired moieties upon formation promotes proliferation, survival, secretion of trophic factors, and differentiation. This approach can be further combined with scaffold‐based constructs to improve delivery and instruct spheroid behavior.	[197, 198]
	Cell sheet	Constructs made of an intact cell layer that preserves ECM by eliminating the use of proteolytic enzymes to harvest the cells, instead using temperature‐sensitive culture systems that allow the release of cells from the substrate after expansion. These sheets can be decellularized to produce dECM sheets for use as a cell culture substrate or coating of 3D scaffolds.	[199, 200]
	3D bioprint	Additive manufacturing technologies that dispense a suspension of cells and proteins (referred to as bioink) under computer guidance. Bioinks can be composed of polymers, natural proteins, or cell‐ or tissue‐secreted dECM. This approach offers material tunability and recapitulation of tissue heterogeneity, as two or more bioinks can be used containing different cell types and dECM.	[201]

### 4.2 Bone Regeneration

Bone is a dense tissue mainly composed of mineralized ECM secreted by osteoblasts. Bone‐derived ECM contains 90% collagen type I, with the remaining 10% consisting of non‐collagenous proteins.^[^ ^136^ ^]^ The osteoconductive properties of demineralized bone matrix were first described in the early 1960s.^[^ ^137^ ^]^ Since then, incorporation of inorganic compounds inspired by bone composition (hydroxyapatite or calcium phosphate‐based particles) remains a common approach to improve osteoconductivity and increase cell attachment in TE scaffolds.^[^ ^138^ ^]^ Currently, the use of bovine bone‐derived dECM has shown promising results as an alternative to patient autologous grafts.^[^ ^139^, ^140^ ^]^ Clinical studies have reported new bone formation for autologous MSCs cultivated on bovine bone‐derived dECM scaffolds. These encouraging results led to the development of several FDA‐approved animal bone‐derived dECM products including PuraGraft, Accell Connexus, Puros DBM, Grafton, BioSet DBX, and Progenix Plus. Human bone‐derived dECM products such as MatrixCellect, Graftys HBS, and OsteoAMP have shown good efficacy as fillers and bone grafts, albeit with a higher production cost compared to animal‐derived products.^[^ ^141^ ^]^ Nonetheless, the widespread use of tissue‐derived dECM is limited by risk of immune response, high batch‐to‐batch variability, and poor host remodeling and integration, thereby slowing their clinical implementation.

Cell‐secreted dECM offers a more versatile alternative to conventional bone‐derived dECM by providing a similar biochemical and structural environment. However, harsh decellularization methods together with 2D and static‐culture conditions have slowed the regenerative potential of dECM for bone regeneration. In recent years, MSC‐secreted ECM has gained momentum for bone regeneration, and dECM has been applied in several approaches to improve synthetic polymeric scaffolds or as a scaffold‐free approach. The superior osteoconductivity of MSC‐secreted dECM is normally attributed to endogenous growth factors (e.g., BMPs, vascular endothelial growth factor), rich collagen content, and fibronectin, and has enabled its use on electrospun scaffolds, cell spheroids, bioprinting, and hydrogel systems. Additionally, dECM secreted by naïve MSCs or cells undergoing osteogenic differentiation can yield substrates that promote tissue mineralization and enhance angiogenic potential. Application of such matrices represent a promising strategy to combat predisposition of implanted osteo‐induced MSCs to undergo apoptosis or to revert phenotype. For instance, the introduction of dECM can significantly improve survival and retention of the osteogenic phenotype in BMSCs.^[^ ^142^ ^]^ However, other factors such as donor age must be considered when producing osteoinductive matrices, as BMSCs from elderly patients secrete ECM with diminished proliferative capacity and osteogenic potential.^[^ ^143^ ^]^

Alternative sources of dECM have also been explored for bone regeneration. For instance, human osteoblasts can secrete instructive ECM that possesses a pro‐regenerative secretome similar to that of MSCs while presenting biochemical cues (e.g., bone sialoprotein, osteopontin, osteocalcin) that drive tissue mineralization.^[^ ^144^ ^]^ Osteoblast‐derived ECM has been successfully implemented as a substrate to induce osteogenesis in human MSCs and employed in the fabrication of melt electrowriting constructs to study its effects on cell viability and tissue mineralization.^[^ ^145^, ^146^ ^]^ Moreover, incorporating dECM from vascularized tissue in a methacrylated hydrogel and BMP‐2 scaffold increased bone formation due to its affinity for growth factors that improve retention and release kinetics.^[^ ^147^ ^]^ Our understanding of bone development highlights the importance of mimicking the mechanical loading environment of native bone to ensure proper function. Decellularized buccal fat pad‐derived stem cell (BFPdSC) scaffolds supplemented with β‐tricalcium phosphate and formed using a rotating and perfusion bioreactor increased the expression of osteogenic markers in BFPdSCs compared to non‐ECM controls.^[^ ^148^ ^]^ To that end, mimicking the in vivo ECM architecture and composition while providing mechanical stimulation could significantly enhance the osteogenic potential of TE constructs.

### 4.3 Cartilage Regeneration

Cartilage tissue is characterized by a dense ECM, low porosity, and a lack of vasculature, which enables low friction articulating joints that are resilient to cyclic loading.^[^ ^149^ ^]^ Furthermore, cartilage has a low chondrocyte density which secretes an ECM rich in collagen and proteoglycans, with other non‐collagenous proteins and glycoproteins present in lesser amounts. The characteristic heterogeneous structure of cartilage, coupled with difficulties in effectively guiding the phenotype of cells under chondrogenic differentiation, necessitates a different approach to generate implantable, functional engineered cartilage. The use of ECM with high collagen content would only recapitulate certain aspects of the deep and calcified zones, as collagen concentrations decrease in the deep layers of articular cartilage. It has been theorized that omitting the characteristic proteoglycan composition and collagen distribution of cartilage ECM (including the presence of collagen II) in TE scaffolds is one cause of chondrocyte dedifferentiation and formation of fibrocartilage.^[^ ^150^, ^151^ ^]^ To overcome this challenge, chondrogenic ECM‐based constructs have relied on the addition of dECM derived from articular cartilage, which has the advantage of preserving native structure and composition.^[^ ^152^ ^]^ Although this strategy enabled successful expansion of chondrocytes and formation of viable cartilage constructs, its reliance on tissue availability limits its clinical translation to date.

Compared to other cell types, chondrocytes exhibit a low proliferative rate in vitro, and their expansion often leads to loss of the chondrogenic phenotype and expression of fibroblast markers, limiting the production of chondrogenic dECM.^[^ ^153^ ^]^ To address this challenge, chondrocytes were cultured on dECM secreted by articular cartilage‐derived chondrocytes, which resulted in improved chondrogenic phenotype and reduced dedifferentiation.^[^ ^154^ ^]^ The capacity of iPSCs to proliferate indefinitely motivates their examination for the generation of chondrogenic constructs and circumventing the limitations associated with somatic chondrocytes.^[^ ^155^ ^]^ Furthermore, the use of human iPSC‐derived chondrocytes represents a promising alternative to create TE constructs for cartilage regeneration.^[^ ^156^ ^]^ However, the mechanisms by which iPSC‐derived chondrocyte dECM instructs cartilage regeneration or differences in composition or bioactivity between somatic and iPSC‐derived chondrocyte dECM remain unknown.

As with bone tissue engineering, MSCs are under widespread investigation for their efficacy in cartilage TE due to their potent secretome and chondrogenic differentiation potential. MSC‐secreted ECM was mixed with 4% agarose to make a composite hydrogel for chondrogenesis.^[^ ^157^ ^]^ Collagen and GAG content were higher for hydrogels with a 2:1 ratio of agarose:ECM, yet the chondrogenic phenotype was better preserved when chondrocytes were entrapped in hydrogels formed with a 1:1 ratio. These data emphasize the importance of balancing mechanical strength and bioactivity for dECM hydrogels. In another example, chondrocyte‐secreted dECM was deposited on poly(ε‐caprolactone) electrospun matrices to instruct MSC chondrogenic differentiation.^[^ ^158^ ^]^ The deposition and bioactivity of ECM were increased when chondrocytes were cultured under low fluid shear stress. Moreover, TGF‐β1 signaling, a potent chondroinductive cue, was potentiated for MSCs when engaging dECM‐coated meshes compared to uncoated meshes. Collectively, these reports demonstrate the promise of dECM to drive cartilage formation using progenitor cells, yet the field must address the variability in ECM content that may influence the efficacy of this approach.

### 4.4 Muscle and Cardiovascular Applications

Muscle is one of several anisotropic tissues with distinct spatial organization of its ECM and residing cells. In order to achieve this orientation, ECM scaffolds containing microchannels have been fabricated by subcutaneously implanting sacrificial templates and decellularizing the explanted tissues to achieve spatial organization and instruct directional migration.^[^ ^159^ ^]^ This novel approach aims to engineer the desired architecture prior to decellularization and thus minimize loss in bioactivity due to scaffold fabrication steps. Due to the highly aligned nature of muscle tissue, several groups developed specific decellularization methods for the preservation of collagen architecture and mechanical properties.^[^ ^160^ ^]^ Recently, the responsiveness of fibro‐adipogenic progenitor cells (FAPs) to changes in matrix stiffness and architecture was explored using dECM from muscle cells in culture.^[^ ^161^ ^]^ These results reveal one potential mechanism for how FAPs support skeletal muscle development and their role in the dysregulation of ECM deposition in muscle pathologies.

Fibrous scaffolds are often used to recapitulate the anisotropic behavior of muscle fibers. Electrospinning of dECM is a common approach that can be combined with fiber functionalization to increase complexity in the final construct.^[^ ^162^, ^163^ ^]^ The combination of electrospinning with electrospraying has yielded composite fibrous scaffolds for small‐scale muscle flaps.^[^ ^164^ ^]^ Alternatively, dECM‐based bioinks have been used to recapitulate the native heart tissue structure in vitro. For example, porcine heart‐derived dECM was deposited on a poly(ethylene/vinyl acetate) frame and cultured under dynamic conditions to enhance cell differentiation and maturation of rat cardiomyocytes.^[^ ^165^ ^]^ Similarly, cardiac‐derived dECM was used to fabricate pre‐vascularized patches with cardiac progenitor cells, resulting in improved vascularization and decreased negative remodeling.^[^ ^166^ ^]^

Similar to cartilage, cardiovascular tissue represents a challenge for TE due to its hierarchical structure and the low proliferative capacity of cardiac cells. The large number of cells required for most bioprinting approaches is a key constraint for the successful fabrication of viable and functional cardiac constructs. Thus, iPSC‐derived cell populations have been incorporated to print cardiovascular constructs with multiple cell types and defined structures. Tissue‐matched dECM bioinks improved the maturation and viability of iPSC‐derived cardiomyocytes while instructing microenvironment organization into striated structures. These findings further support the significance of dECM substrates in providing a conducive environment for cell maintenance^[^ ^167^ ^]^ and emphasize the importance of tissue sources for dECM.

### 4.5 Vascularization

The potential to construct large tissue engineered constructs is limited by the presence of a functional vasculature. Construct viability is dependent upon sufficient nutrient transport to the resident cells. To this end, dECM enriched in proangiogenic factors is an important platform for vascular reconstruction and angiogenesis. The basement membrane (collagen IV, fibronectin, heparan sulfate, and proteoglycans) and interstitial ECM (collagen I and elastic fibers) are the main components of the vascular ECM. Although both have been studied individually for their angiogenic potential, the basement membrane plays a key role in endothelial cell attachment, proliferation, migration, and angiogenic activity.^[^ ^168^, ^169^ ^]^ In an effort to balance effective decellularization while preserving the bioactivity of basement membrane‐derived ECM,^[^ ^170^ ^]^ studies revealed that harsh detergent‐mediated decellularization decreased both collagen and GAG content, thereby limiting endothelial cell infiltration and inducing an aberrant phenotype. These findings have broad implications on the retention of endogenous growth factors within the dECM and its bioactivity for numerous applications.

Tissue‐derived ECM vascular grafts modulate adhesion, proliferation, and migration of endothelial cells due to the preservation of vascular structure.^[^ ^171^ ^]^ In contrast, the use of cell‐secreted ECM has been commonly employed as a substrate to improve vascularization of TE scaffolds. Vessel‐derived ECM was harnessed for growth factor delivery and vascularization when delivering BMP‐2 for osteogenic regeneration. Furthermore, HUVEC‐derived dECM promoted endothelial‐lineage differentiation of progenitor cells derived from exfoliated deciduous teeth.^[^ ^172^ ^]^ The duality of ECM as a delivery carrier while instructing tissue regeneration was further demonstrated by introducing a complex protein network from decellularized ovine aortas to deliver BMP‐2.^[^ ^147^ ^]^ Overall, these studies demonstrate the capacity of dECM to improve different aspects of tissue regeneration in cell‐based scaffolds

## 5 Future Outlook

The development of dECM‐based platforms has progressed rapidly over the last two decades. Current decellularization methods are superior in preserving composition and structure while being inexpensive and highly translatable. However, cell sourcing, rate of ECM production, deployment, and potential immune response represent ongoing limitations of ECM products for clinical translation. ECM production and cell sourcing may be overcome with the emergence of iPSC‐derived cell populations as a viable source for cell‐based therapies.^[^ ^173^ ^]^ Nonetheless, the immunomodulatory response, degradation, and remodeling processes of dECM‐based constructs have not been thoroughly tested. To capitalize on the attributes of iPSCs and their instructive ECM, it is critical for allogeneic iPSC‐derived dECM and associated byproducts during degradation to undergo thorough characterization to support clinical translation.

The evolution of decellularization methods has resulted in safe and reproducible techniques that ensure the preservation of ECM composition with some acceptable reduction in bioactive potential. However, novel decellularization methods that are capable of removing cellular components while enhancing the regenerative potential of the dECM scaffold can lead to the development of constructs that benefit from a specific host response. For example, dECM that can mediate ROS and reactive nitrogen species (RNS) has shown promising results regarding improved cell proliferation, viability, protection from oxidative damage, and anti‐inflammatory response.^[^ ^174^ ^]^ Bioorthogonal chemistry also promises to improve our understanding of the mechanism by which dECM constructs instruct tissue regeneration by labeling, visualization, and quantification of ECM components.^[^ ^175^ ^]^ This approach could be used to modify secreted ECM and instruct specific protein–cell interactions. Emerging gene editing techniques represent an exciting future for dECM‐based constructs. For example, genetic modification of ECM‐secreting cells could enable the production of tailored matrices, increased content of growth factors or other bioactive compounds, and the capacity to create ECM‐compositions that better mimic heterogeneous tissues.^[^ ^176^ ^]^ For example, the study of deleterious mutations in skin ECM motivated clinical trials examining the transplantation of transgenic keratinocyte cultures that can regenerate fully functional epidermis for the treatment of epidermolysis bullosa using retroviral transduction of the LAMB3 laminin‐encoding gene.^[^ ^177^ ^]^

As the matrisome includes more than just matricellular proteins and glycosaminoglycans, it is essential to strive for a more thorough characterization of the dECM. Extracellular vesicles (EVs) are potent mediators in cell signaling, tissue homeostasis, immune response, inflammation, and angiogenesis.^[^ ^178^, ^179^ ^]^ Matrix‐bound EVs are key regulators in several commercially available and laboratory‐produced dECM scaffolds, which contain microRNA that can instruct cell behavior.^[^ ^22^ ^]^ Matrix‐bound EVs can be specifically engineered to bind to the underlying matrix, providing a unique opportunity to deliver this bioactive payload in a controlled, intentional manner.^[^ ^180^ ^]^ A deeper understanding of the role of matrix‐bound EVs in the modulation of cell behavior could alter how we ascribe dECM bioactivity and lead to the development of improved dECM processing techniques. Although the mechanism between EV transport and function and dECM remains elusive, the role of dECM on the regenerative potential of the progenitor cell secretome has been interrogated for its applications in drug delivery.^[^ ^181^ ^]^ This work shows the capacity of dECM to improve the therapeutic potential of soluble cues naturally secreted by progenitor cells, making dECM‐constructs a promising platform for delivery of bioactive factors.

**Figure 5:**
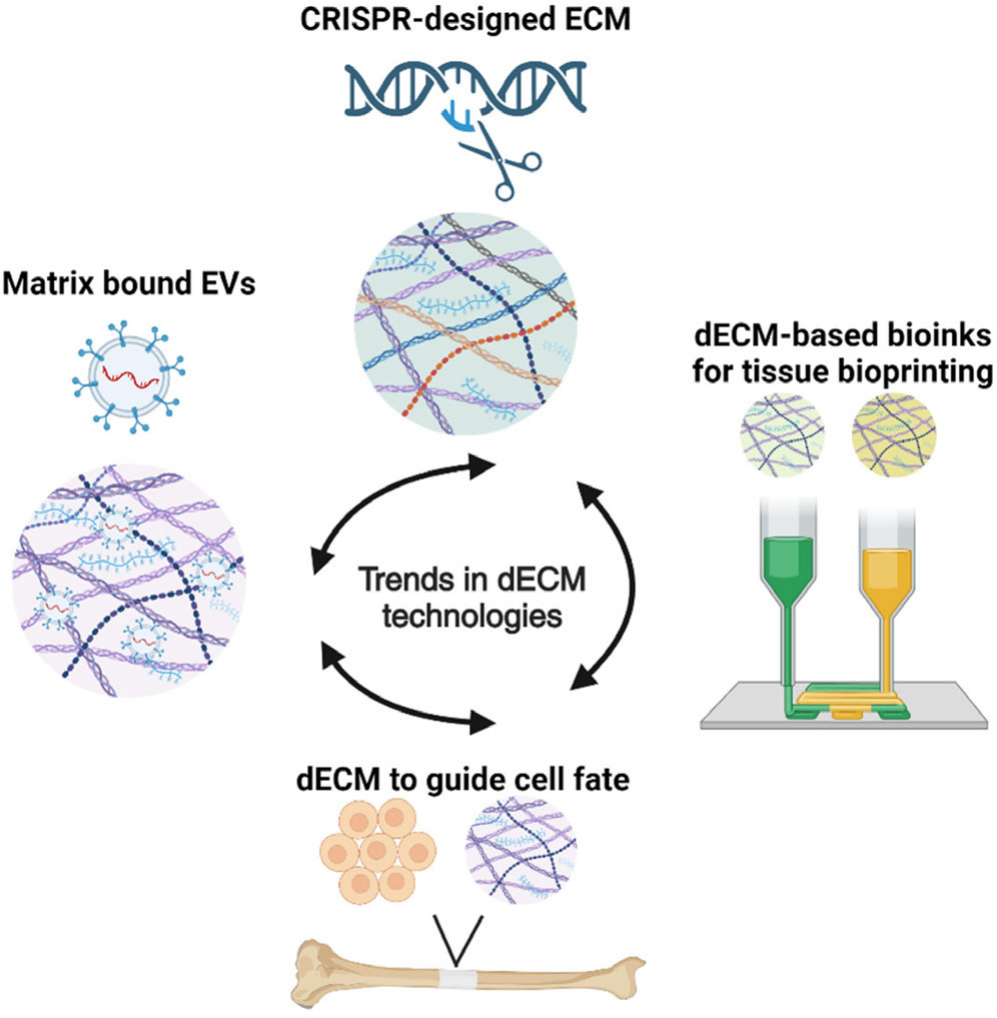
Current trends and technologies to improve dECM‐based platforms. Developments in bioink formulation and genetic modification (e.g., CRISPR) may enable fabrication of dECM constructs. Meanwhile, our understanding of bioactive elements within the dECM, such as matrix‐bound EVs, will improve our current approaches on how to guide cell fate using dECM constructs without the need for additional bioactive soluble factors.

The implementation of microfabrication techniques for control of topographical cues may facilitate the production of highly specific dECM to meet requirements in cell alignment and organization, thus closely mimicking the heterogeneous nature of tissue‐derived ECM. Similarly, the development of dECM‐derived bioinks as an alternative to individual proteins or polymers may yield more complex 3D constructs that possess similar biochemical and mechanical properties to native tissue.^[^ ^182^ ^]^ dECM‐based granular hydrogels (microgels) may also provide a platform to circumvent challenges of decoupling mechanical properties and bioactivity for dECM constructs. Tissue‐derived dECM microgels were used to create bioinks with improved printability and mechanical properties versus conventional dECM‐based inks while improving endothelial and Schwann cell viability.^[^ ^183^ ^]^ **Figure** **5** shows a schematic representation of current trends for dECM‐based tissue engineering.

## 6 Conclusion

The importance of the ECM in regenerative medicine cannot be overstated, not only for its role in providing adhesive sites for cells and mechanical integrity but also for instructing cell function through its composition and biophysical properties. The use of individual proteins to form constructs that mimic ECM composition cannot fully recapitulate the dynamic relationship of soluble cues and structural moieties present within the native ECM. Thus, developments in the utilization of dECM may provide an improved strategy to recreate the native cell microenvironment and increase the regenerative potential of conventional polymeric scaffolds.

The bioactivity of dECM can be improved by careful selection of cell sources, modulating culture conditions, and functionalizing dECM to stimulate specific cell behavior according to the desired applications. Nonetheless, the translational potential of dECM remains limited due to its availability, reproducibility, and over‐reliance on incomplete or damaging decellularization methods. iPSCs, when used as a source for cell‐secreted ECM, may offer scalability and potentially decrease batch‐to‐batch variations that hinder the clinical translation of dECM. New approaches are necessary to reproducibly control the mechanical properties and degradation of dECM without decreasing its natural bioactivity. The identification of cost‐effective and reliable approaches to reproducibly produce, characterize, sterilize, and deploy dECM will provide a valuable tool in creating or repairing damaged tissues.

## Conflict of Interest

The authors declare no conflict of interest.

## Author Contributions

**David H. Ramos‐Rodriguez**: conceptualization (supporting); data curation (lead); formal analysis (lead); funding acquisition (equal); writing—original draft (lead); writing—review and editing (equal). **J. Kent Leach**: conceptualization (lead); funding acquisition (equal); project administration (lead); supervision (lead); writing—review and editing (lead).
